# Construction of a novel miRNA regulatory network and identification of target genes in gestational diabetes mellitus by integrated analysis

**DOI:** 10.3389/fgene.2022.966296

**Published:** 2022-12-01

**Authors:** Liyan Ding, Yi Shen, Anqi Wang, Changlian Lu, Xuefeng Gu, Liying Jiang

**Affiliations:** ^1^ Department of Epidemiology, School of Public Health, Nantong University, Nantong, Jiangsu, China; ^2^ Department of Nursing, Collaborative Research Center, Shanghai University of Medicine & Health Sciences, Shanghai, China; ^3^ Shanghai Key Laboratory of Molecular Imaging, Zhoupu Hospital, Shanghai University of Medicine and Health Sciences, Shanghai, China; ^4^ School of Pharmacy, Shanghai University of Medicine & Health Sciences, Shanghai, China; ^5^ Shanghai Key Laboratory of Molecular Imaging, Jiading Central Hospital, Shanghai University of Medicine and Health Sciences, Shanghai, China

**Keywords:** GDM, MicroRNAs, RNA-seq, bioinformatic analysis, peripheral blood leukocytes

## Abstract

**Backgrounds:** Given the roles of microRNA (miRNA) in human diseases and the high incidence of gestational diabetes mellitus (GDM), the aim of the study was to examine miRNA signatures and crucial pathways, as well as possible biomarkers for GDM diagnosis.

**Methods:** We conducted a two-stage study to explore functional miRNA and those target genes. Twelve participants (6 GDM and 6 non-GDM) were first enrolled and performed RNA sequencing analysis. The overlapped candidate genes were further screened in combination with differentially expressed genes (DEGs) of GEO datasets (GSE87295, GSE49524 and GSE19649) and potential target genes of DEMs. Candidate genes, critical pathways, small molecular compounds and regulatory networks were identified using bioinformatic analysis. The potential candidate genes were then investigated using the GEO dataset (GSE103552) of 19 participants in the validation stage (11 GDM and 8 non-GDM women).

**Results:** Briefly, blood samples were sequenced interrogating 50 miRNAs, including 20 upregulated and 30 downregulated differentially expressed microRNAs(DEMs) in our internal screening dataset. After screening GEO databases, 123 upregulated and 70 downregulated genes were overlapped through DEGs of GEO datasets and miRNA-target genes. MiR-29b-1-5p-TGFB2, miR-142-3p-TGFB2, miR-9-5p-FBN2, miR-212-5p-FBN2, miR-542-3p-FBN1, miR-9-5p-FBN1, miR-508-3p-FBN1, miR-493-5p-THBS1, miR-29b-3p-COL4A1, miR-432-5p-COL5A2, miR-9-5p-TGFBI, miR-486-3p-SLC7A5 and miR-6515-5p-SLC1A5 were revealed as thirteen possible regulating pathways by integrative analysis.

**Conclusion:** Overall, thirteen candidate miRNA-target gene regulatory pathways representing potentially novel biomarkers of GDM diseases were revealed. Ten chemicals were identified as putative therapeutic agents for GDM. This study examined a series of DEGs that are associated with epigenetic alternations of miRNA through an integrated approach and gained insight into biological pathways in GDM. Precise diagnosis and therapeutic targets of GDM would be further explored through putative genes in the future.

## Introduction

Gestational diabetes mellitus (GDM) is a comprehensive form of pregnancy-specific glucose intolerance or hyperglycemia that manifests certain degree of glucose intolerance (Plows et al., 2018). Hyperglycemia occurs in approximately 16.7% in pregnancies worldwide, 75%–90% of which is caused by GDM, implying that GDM has become a significant public health concern (IDA, 2021). Although blood glucose level in GDM usually returns to normal after delivery, women are at high risk of acquiring type 2 diabetes later in life, and the risk of metabolic syndrome and insulin resistance in offspring have also increased (Damm et al., 2016), which presents a vicious intergenerational cycle. Compelling evidence suggest that advanced maternal age, family history of diabetes, diet, physical activity or emerging environmental factors are likely to have an impact on the risk of developing GDM (Zhang et al., 2016). However, knowledge concerning the detailed processes governing the initiation and progression of GDM remains unknown.

Although Genome-wide association studies (GWAS) have revealed several genetic loci correlated with the complexity of the disease, the underlying mechanism remain unclear (Kwak et al., 2012). Non-coding RNAs (ncRNAs) are important players in metabolic processes and their deregulated expressions have been observed in several metabolic diseases, including GDM. MicroRNA (miRNA), as a type of generally ubiquitous and multifunctional short non-coding RNAs with 19–22 nucleotides that regulate post-transcriptional gene expression (Brennecke et al., 2005), and participate in a range of biological functions (Du and Zamore, 2005).

Currently, in spite of the presence of discordant data, numerous researches indicate that circulating miRNAs are involved in mediating the key pathophysiological features of GDM, including glucose homeostasis, inflammation, insulin resistance, metabolic adaptations and β-cell dysfunction (Sliwinska et al., 2017; Filardi et al., 2020; Abu Samra et al., 2022). Aberrant levels of miRNA and gene expression in GDM have laid a favorable foundation for personalized target therapy and potential drugs. Compelling studies show that miR-195-5p overexpression in GDM women play an important role in insulin insensitivity regulation (Tagoma et al., 2018; Wang et al., 2020). A study of Filardi et al. identified that miR-222-3p and miR-409-3p signatures were significantly up-regulated in GDM, which are correlated with fasting plasma glucose (Filardi et al., 2022). Concerted efforts are made to gain knowledge about the mechanisms by which metabolic pathways are coordinated by acquired and genetic factors to explore novel insights into GDM treatment.

Although common biomarkers for GDM have been identified previously, the specific molecular mechanisms remain unclear. Genetic susceptibility is represented by gene variants conferring individual differences in response to metabolic-related chronic diseases. Most importantly, few studies have used transcriptome sequencing to explore GDM biomarkers, especially in South-East Chinese population. To the best of our knowledge, systematic regulatory network and pathways have not been well profiled about how alternations of microRNAs and mRNA are related to GDM development integrating with chemical compounds. The understanding of their functions might help unravel the complex pathophysiological mechanisms and identify novel clinical treatment. Early prevention and diagnosis are of great important to avoid adverse effects.

In this present study, a complete transcriptome RNA based on GDM in peripheral blood leukocytes (PBL) was sequenced, and putative hub genes were identified using a stepwise screening approach. The target genes of differentially expressed miRNA (DEMs) were synchronously predicted *via* online miRNA-target databases. The transcriptome were utilized to reveal molecular pathways and protein-protein interaction (PPI) network of candidate genes and confirmed those hub genes, followed by an external validation on the expression levels through the GSE103552 dataset (11 GDM and 8 normal controls). Simultaneously, by querying through CMap, registered chemicals could be screened and the likelihood of drugs could be obtained, showing gene expression profile that discover potential small molecules targeting diseases. This study aimed to identify pivotal pathways for complex pathophysiological mechanisms for GDM and contextually explore novel potential diagnostic biomarkers for the disease.

## Materials and methods

### Sample collection

In the first RNA-sequencing screening stage, blood samples were collected form 6 GDM from the Nantong Maternal and Child Health Hospital (NMCHH) in October 2020, and in the meantime 6 GDM-free women were randomly selected from a pool of more than 100 individuals who participated in routine healthcare examination in the same hospital. Non-GDM women were matched with GDM cases according to the age.

The methods for diagnosing GDM were mentioned in our study previously (Shen et al., 2020). Those participants conform to the diagnosis criteria of GDM [International Association of Diabetes Pregnancy Study Group (IADPSG)] plus complete demographic information recruited (Metzger et al., 2010). GDM patients with complications such as diabetes mellitus, chronic hypertension, pre-eclampsia and inflammatory diseases were excluded.

The study was reviewed and approved by the Ethics Committee of Shanghai University of Medicine & Health Sciences. All participants signed written informed consent.

### Library preparation and sequencing

White blood cells were extracted by centrifugation at 1,500 g for 20 min with 2 ml whole blood. We utilized TRIzol (Invitrogen, Carlsbad, CA, United States ) to extract total RNAs following the manufacture’s protocol.

YueDa Biotechnology Co., Ltd. (Shanghai, China) performed RNA-seq using 150 ng of total RNA as input, and the results were analyzed by an Illumina HiSeq 2,500 sequencing platform with 10 M reads (Illumina, San Diego, CA, United States ). Bowtie were used to compare DEMs (Langmead, 2010), and feature Counts were adopted to annotate and quantify miRNAs (Liao et al., 2014). Counts were assessed and DEMs were filtered using the DESeq2 package in R (http://bioconductor.org/packages/release/bioc/html/DESeq2.html). Herein, miRNAs with fold changes (FC) > 1.5 or <0.667 and *p* < 0.05 were considered as significance.

### Prediction of downstream target genes of DEMs

Target genes were concurrently predicted using Targetscan 7.2 (http://www.targetscan.org/vert_72/), miRDB (http://mirdb.org/), and miRwalk (http://mirwalk.umm.uni-heidelberg.de/) based on the aforementioned main DEM analysis. The anticipated target genes that better fit among three databases were regarded as those target genes of DEMs for a really steady selection.

### Data collection and identification of overlapped candidate genes

In this present study, mRNA expression profiling datasets by array (GSE87295, GSE49524 and GSE19649) were retrieved and obtained from the GEO database (Gene Expression Omnibus, https://www.ncbi.nlm.nih.gov/geo/). Of these, 5 HUVECs GDM samples and 5 HUVECs controls were enrolled in GSE87295 (platform: GPL10558); 3 Caucasian Gestational diabetes women and 3 Caucasian non diabetic women in GSE49524 (platform: GPL7020); 3 GDM and 2 control in GSE19649 (platform: GPL7350).

We identified differentially expressed genes (DEGs) for each of three datasets separately by using linear models for microarray (LIMMA) approach through GEO2R online tool (https://www.ncbi.nlm.nih.gov/geo/geo2r/). The screening thresholds for promising DEGs were set at *p* < 0.05 and fold change (FC) > 1.5 or <0.667. Subsequently, DEGs and potential target genes of DEMs were overlapped to obtain candidate genes. Herein, those genes of intersection between two datasets would be considered as being included. The ggVennDiagram software package in R (https://cran.r-project.org/web/packages/ggVennDiagram/index.html) was used to plot the Venn diagrams.

### Functional and pathway enrichment analysis

Gene ontology (GO) analysis and Kyoto Encyclopedia of Genes and Genomes (KEGG) pathway enrichment analysis were conducted for candidate genes by DAVID v6.8 (Database for Annotation, Visualization and Integrated Discovery) (https://david.ncifcrf.gov/home.jsp). *p* < 0.05 for GO analysis, and *p* < 0.1 and count>2 for the KEGG were considered for further analysis.

### Candidate small molecules discovery in CMap

We used Connectivity Map (CMap) to explore potential therapeutic agents related to GDM. CMap database (https://clue.io/query) is an open database that predict those potential small molecular compounds of altered expression of DEGs in cell lines, presenting a connectivity score from -100 to 100. Score closer to 100 indicates that gene list is more similar change to the molecule. Conversely, a negative score indicates that small molecular compounds express antagonism, which could be candidate molecules for the treatment of GDM.

### Protein-protein interaction network construction and screening of hub genes

The investigation of protein-protein interaction (PPI) network is crucial in assessing the disease’s molecular process. The Search Tool for the Retrieval of Interacting Genes (STRING) online tool (http://stringdb.org/) was employed to construct a network of candidate genes. The node pairs with a combined score of less than 0.4 were selected for further exploration. The network was visualized using Cytoscape v3.9.1, and hub genes were screened according to degree using CytoHubba, a Cytoscape plugin. The Maximal Clique Centrality (MCC) method was used to choose the top 30 target genes.

### Expression analysis of hub genes based on GSE103552

The GSE103552 database [platform GPL6244; 11 GDM and 8 normal foetoplacental arterial endothelial cells (AEC)] was used to examine the expression level of hub genes for external verification. The criteria for determining hub genes also definitely are consistent with the previous demonstration.

### Statistical analysis

Continuous variables adhering to the normal distribution were represented as the mean ± standard deviation; otherwise, the interquartile range (P_25_-P_75_) was substituted. The difference of continuous variables was tested by the independent sample t-tests or the Mann-Whitney tests. Categorical variables were represented as n (proportion), and the difference were tested by the χ^2^ tests. Body mass index (BMI) was categorized four parts according to the Working Group on Obesity in China recommended criteria (Underweight: BMI < 18.5; Normal: 18.5 ≤ BMI < 24; Overweight: 24 ≤ BMI < 28; and General obesity: BMI ≥ 28 kg/m^2^) (Zhou, 2002). A value of *p* < 0.05 was considered statistically significant. All analysis was performed with SPSS 20.0 (IBM Corp. Chicago, IL) and GraphPad Prime9.0 (GraphPad Software, Inc.).

## Results

**FIGURE 1:**
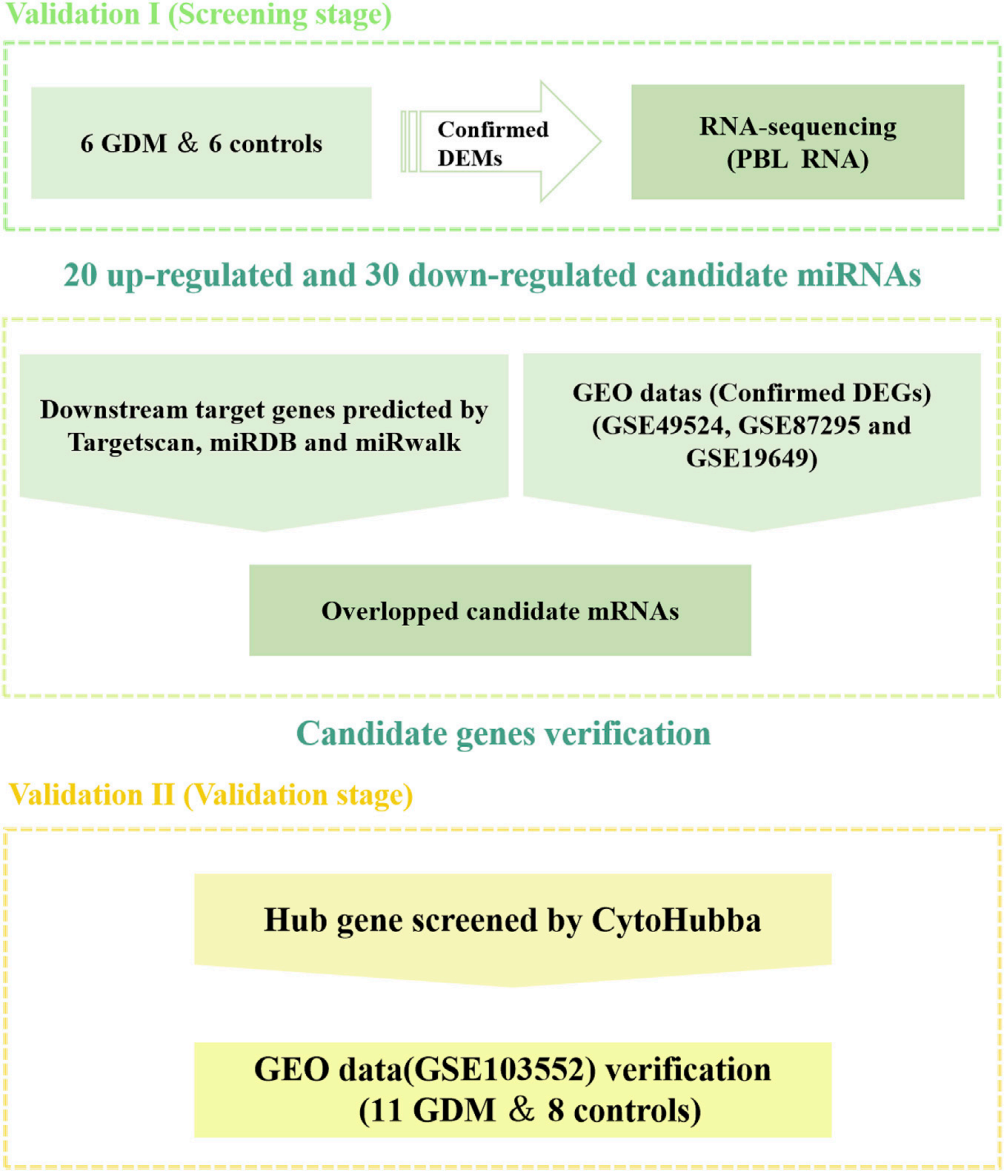
Schematic of study design.

Figure 1 depicts a schematic representation of the study design. And, Table 1 summarizes the characteristics of 12 samples. There was no significant difference between GDM patients and healthy controls in terms of age, BMI, 2h-plasma glucose, systolic blood pressure (SBP) and diastolic blood pressure (DBP) (*p* > 0.05). GDM patients had a statistically significant higher fasting plasma glucose (FPG) and 1h-plasma glucose (*p* < 0.05) as compared to controls.

**TABLE 1:** Characteristics of the subjects enrolled for miRNA expression analysis in the study.

Variables	GDM(*n* = 6)	Normal (*n* = 6)	t/χ^2^	*P*
Age^a^	28.17 ± 4.070	29.50 ± 4.722	−0.524	0.612
BMI				
BMI < 18.5	2 (33.33%)	0	5.351	0.061
18.5 ≤ BMI < 24	2 (33.33%)	6 (100%)		
24 ≤ BMI < 28	1 (16.67%)	0		
BMI ≥ 28	1 (16.67%)	0		
fasting plasma glucose (FPG)^a^	5.16 ± 0.499	4.633 ± 0.268	2.284	0.045
1h-plasma glucose^a^	9.86 ± 0.553	9.15 ± 0.331	2.705	0.022
2h-plasma glucose^a^	7.92 ± 1.861	7.33 ± 0.859	0.699	0.500
systolic blood pressure (SBP)^a^	116.50 ± 12.373	115.83 ± 18.713	0.073	0.943
diastolic blood pressure (DBP)^a^	72.83 ± 8.400	76.00 ± 12.458	−0.516	0.617

^a^ Mean ± SD. BMI, body mass index, kg/m^2^.

### Identification and prediction of target genes of DEMs

**FIGURE 2:**
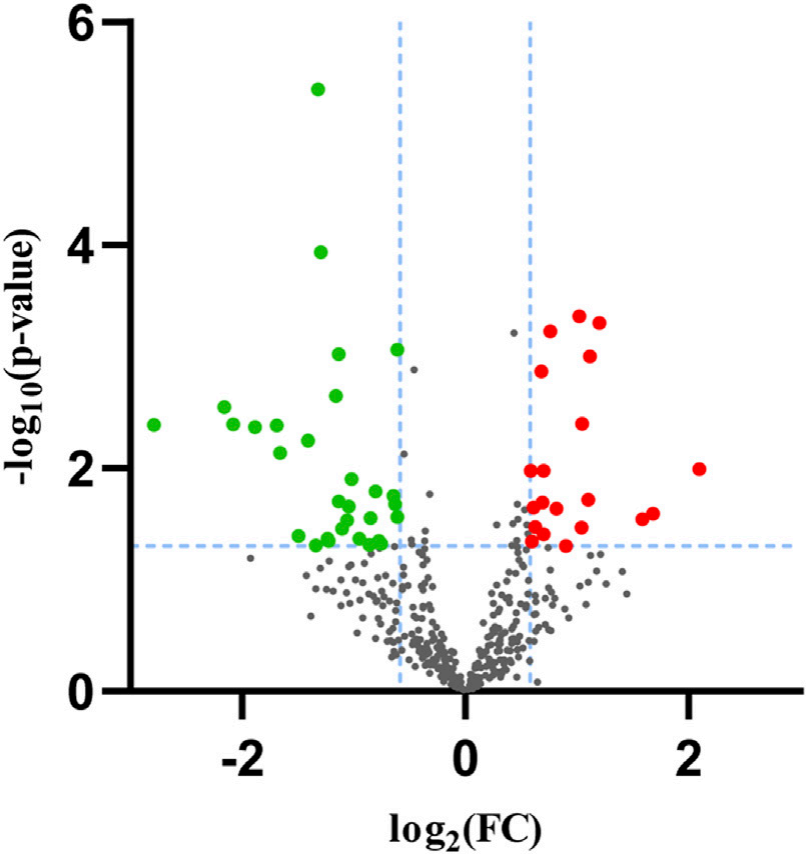
The volcano map of differentially expressed miRNAs(DEMs).

In our sequencing dataset, a total of 20 upregulated and 30 downregulated miRNAs were explored and identified based on the selection standard of *p* < 0.05 and fold change (FC) > 1.5 or < 0.667. The volcano map of the database was presented in Figure 2. We utilized the web tools TargetScan, miRDB, and miRwalk to explore the overlapped target genes of miRNAs by Venn diagram analysis, as shown in Table2. There were 3,867 target genes for upregulated DEMs and 3,856 target genes for downregulated DEMs after removing those duplicates.

**TABLE 2:** miRNAs’ Fold Change and miRNA-target genes count.

miRNAs	FC	TargetScan	miRDB	miRwalk	Overlapped target genes
Upregulated DEMs					
miR-425-3p	2.029890634	928	35	13464	22
miR-4664-3p	2.062528422	631	36	15671	25
miR-326	2.06518067	322	759	16527	190
miR-1908-5p	2.147315234	2,522	193	16837	164
miR-486-5p	2.166278906	174	331	14788	77
miR-6852-5p	2.300671494	6,611	1,025	17519	899
miR-11401	3.003034398	—	71	14701	59
miR-181b-3p	3.20666357	182	743	2017	155
miR-3127-3p	4.283048458	2,396	513	16497	441
miR-7976	1.869581029	4,274	553	11229	414
miR-3177-3p	1.762529006	978	65	16173	49
miR-345-5p	1.695004378	3,528	319	15989	253
miR-486-3p	1.62752759	5,264	942	16993	785
miR-378a-5p	1.62647427	5,497	653	16198	549
miR-151a-3p	1.618792421	112	220	10460	27
miR-484	1.602668449	1755	498	15281	117
miR-652-3p	1.544724973	17	16	14531	4
miR-500a-3p	1.529596365	3,073	292	15068	231
miR-6515-5p	1.5130105	4,797	597	17498	527
let-7d-3p	1.504693869	490	44	11982	20
Downregulated DEMs					
miR-516b-5p	0.144798243	5,175	698	11289	467
miR-664b-5p	0.224525312	2,242	254	16679	212
miR-29b-1-5p	0.236515559	4,358	695	15213	560
miR-409-5p	0.27075915	136	198	9,747	36
miR-582-5p	0.310145049	637	1,143	4,933	134
miR-301a-3p	0.317139174	191	916	3,473	48
miR-508-3p	0.355768824	2,474	417	11136	273
miR-7-5p	0.377368524	566	875	5,851	185
miR-212-5p	0.396315968	442	494	15018	146
miR-9-5p	0.401244911	1,388	1,236	9,705	601
miR-454-3p	0.408624043	294	956	4,715	71
miR-432-5p	0.425604999	3,902	475	16998	397
miR-375-3p	0.429045334	—	269	12032	192
miR-155-5p	0.448525216	556	701	5,949	157
miR-365a-5p	0.456478242	3,245	243	17737	163
miR-142-3p	0.456699463	—	418	7,695	228
miR-889-3p	0.465748814	4,716	931	627	54
miR-493-5p	0.479890447	796	1,217	10223	330
miR-34c-5p	0.484697988	321	803	14104	158
miR-181d-5p	0.493770996	292	1,408	10976	148
miR-125a-5p	0.519289155	512	921	12340	253
miR-1255a	0.550969361	2,984	369	4,732	109
miR-29b-3p	0.556549534	278	1,034	4,361	88
miR-200c-3p	0.57340723	44	1,244	10486	23
miR-543	0.584496787	757	1,208	9,087	246
miR-542-3p	0.594446138	352	588	2,475	51
miR-19b-3p	0.640893703	132	1,329	4,470	42
miR-548e-3p	0.646705596	519	1,637	2,717	38
miR-196b-5p	0.656379461	72	369	14328	31
let-7f-5p	0.657003518	64	991	7,221	27

FC; fold change.

### Identification of candidate genes

The mRNA expression profiling datasets by array (GSE87295, GSE49524 and GSE19649) were employed to screen DEGs *via* GRO2R and further analyzed the hub genes and pathways. Briefly, 324 DEGs were detected in HUVECs samples from 5 GDM and 5 control, including 180 upregulated and 144 downregulated genes in GSE87295. 334 DEGs were detected in 3 Caucasian GDM women and 3 healthy controls in GSE49524, including 206 upregulated genes and 128 downregulated genes. Meanwhile, 161 DEGs were evaluated in GSE104297, including 109 upregulated mRNAs and 52 downregulated mRNAs(3 GDM and 2 non-GDM).

**FIGURE 3:**
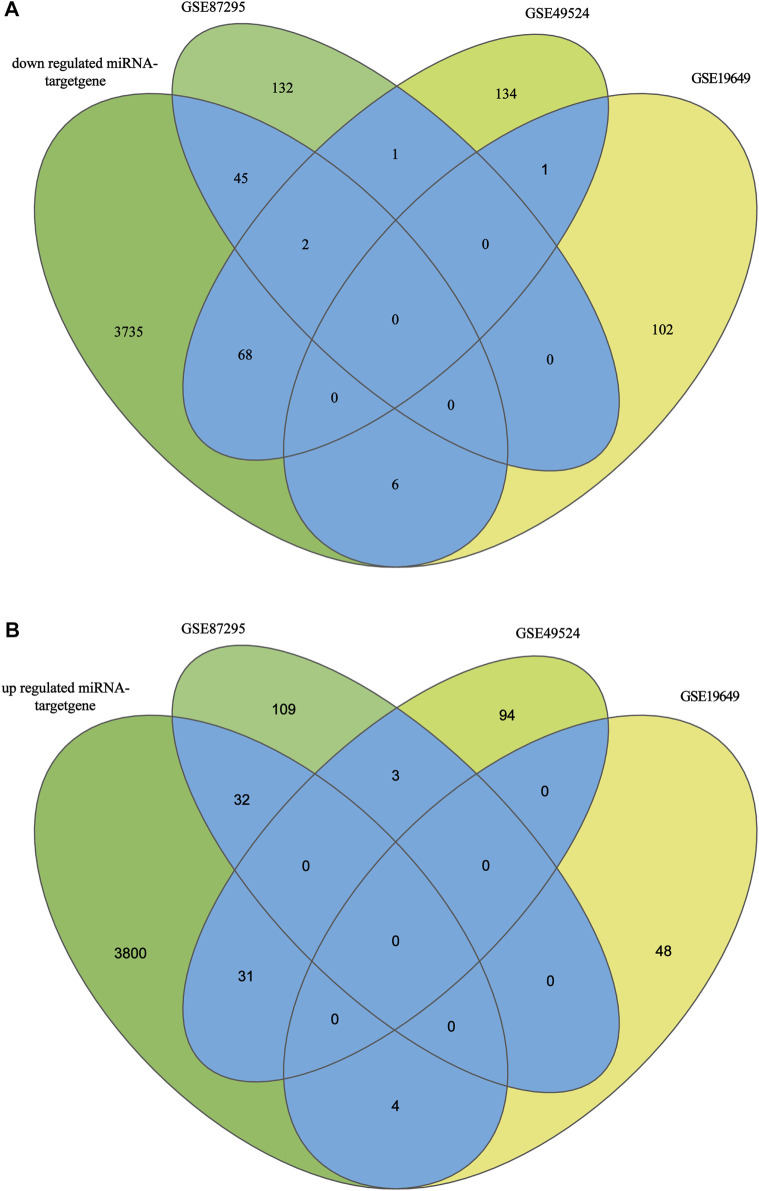
**(A)**: Target genes of low expression miRNAs and upregulated mRNAs of datasets. **(B)**: Target genes of high expression miRNAs and downregulated mRNAs of datasets.

**FIGURE 4:**
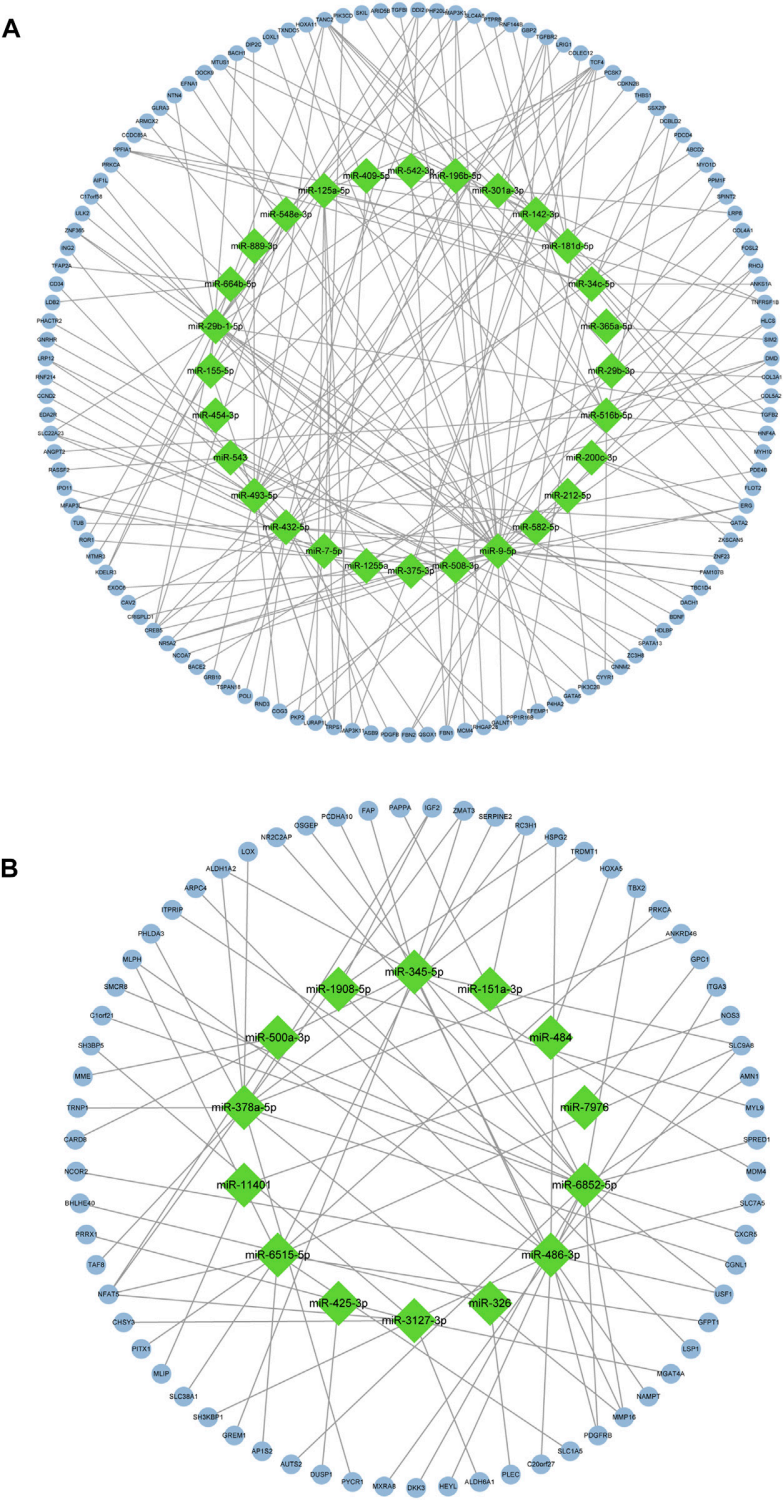
Regulatory network graph. **(A)**: 28 low-expression miRNAs and their target genes. **(B)**: 14 high-expression miRNAs and their target genes.

After screening of internal 12 samples and 3 GEO databases, the comprehensive datasets shared 193 overlapped candidate genes (123 upregulated genes and 70 downregulated genes). The overlapped genes were displayed in Figures 3A,B. The DEMs-gene network was established to analyze the relationship between DEMs and genes intuitively (Figures 4A,B). Among these DEMs, there were 67 target genes for 14 upregulated miRNAs and 121 ones for 28 downregulated miRNAs. Owing to the unavailability of the intersection with DEGs for those remaining miRNAs’ target genes, miRNA-mRNA relationship have not been identified. Furthermore, with a view to 5 genes representing the intersection of two DEGs, miRNAs of these genes were unavailable [ANKRD16, STAT1 (upregulated genes) and IGFBP6, PLAT, PLAC9 (downregulated genes)].

### Enrichment analysis of candidate genes

**FIGURE 5:**
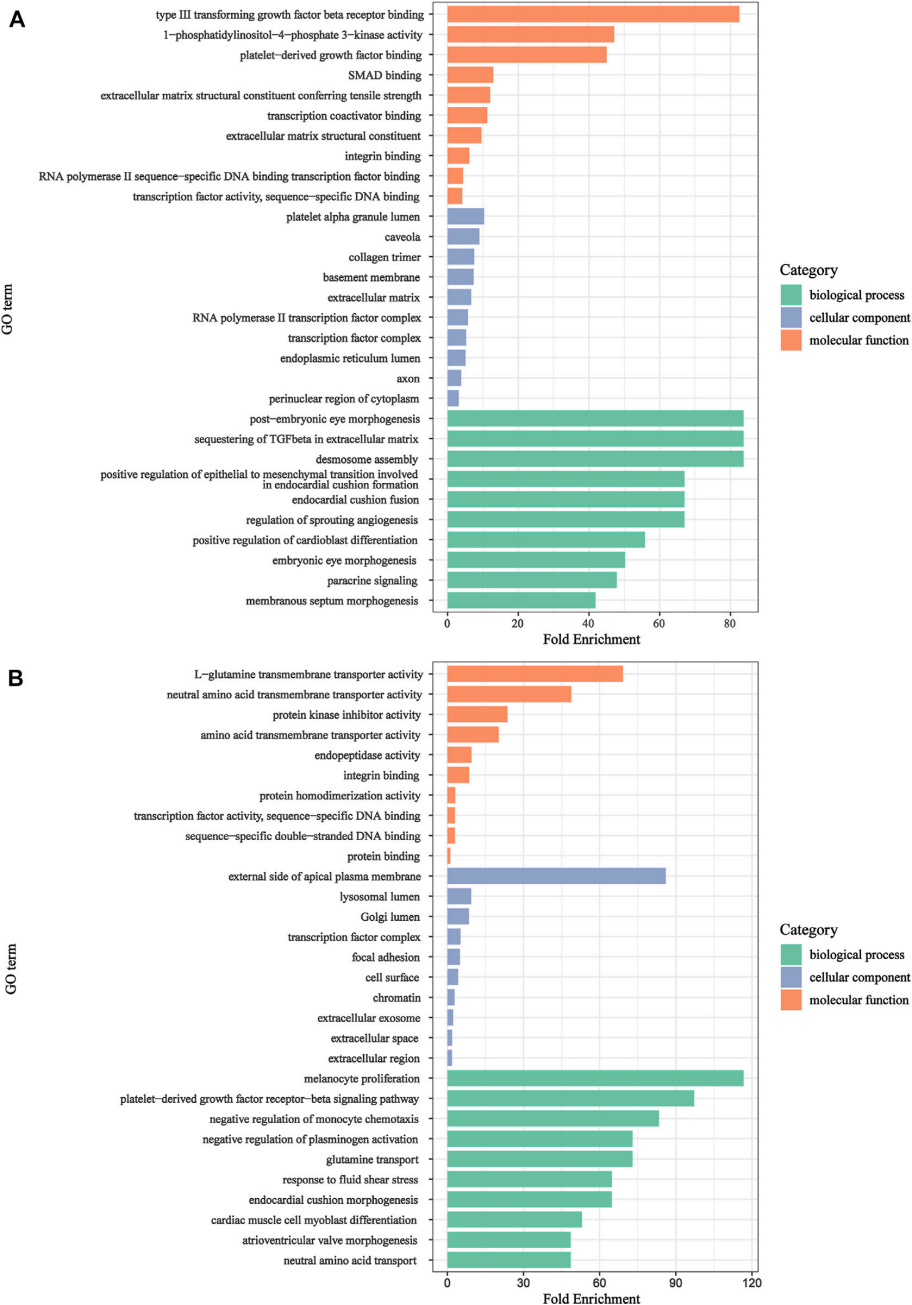
GO annotation analysis for candidate genes in the biological process, cellular component, and molecular function. **(A)**: upregulated genes. **(B)**: downregulated genes.

To explore biological features and enriched pathways of those candidate genes, GO and KEGG analysis were accomplished by DAVID online tools. GO enrichment results were shown in Figures 5A,B, respectively. The upregulated genes were primarily enriched in paracrine signaling, desmosome assembly, sequestering of TGF-β in extracellular matrix, *etc.,* in the BP group; platelet alpha granule lumen, transcription factor complex, caveola, *etc.,* in the CC group; platelet-derived growth factor binding, extracellular matrix structural constituent, type III transforming growth factor beta receptor binding, *etc.,* in the MF group. While, the downregulated genes were mainly involved in glutamine transport, negative regulation of plasminogen activation, melanocyte proliferation, platelet-derived growth factor receptor-beta signaling pathway, *etc.,* in the BP group; external side of apical plasma membrane, focal adhesion, lysosomal lumen, *etc.,* in the CC group; L-glutamine transmembrane transporter activity, neutral amino acid transmembrane transporter activity, amino acid transmembrane transporter activity, *etc.,* in the MF group.

**FIGURE 6:**
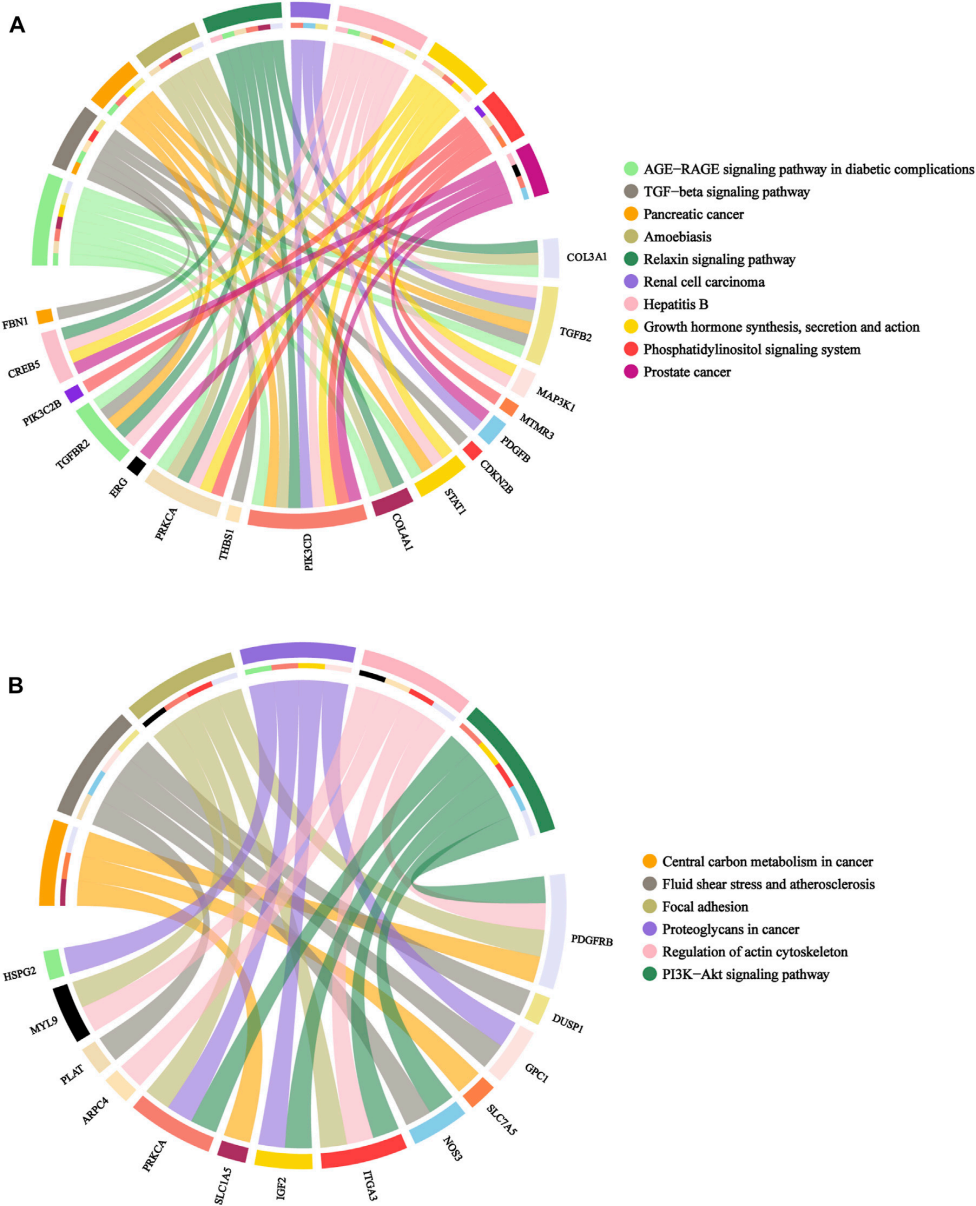
Chord diagram of KEGG pathway analyses for candidate genes. **(A)**: upregulated genes. **(B)**: downregulated genes. Legend: pathway names.

Subsequently, KEGG were conducted to explore the enrichment analysis of these candidate genes. Upregulated genes were mostly associated with pancreatic cancer, TGF-β signaling pathway, AGE-RAGE signaling pathway in diabetic complications, growth hormone synthesis, secretion and action and phosphatidylinositol signaling system, whereas downregulated genes were mainly associated with proteoglycans in cancer, PI3K-Akt signaling pathway, fluid shear stress and atherosclerosis and regulation of actin cytoskeleton, as shown in Figures 6A,B.

### Related small molecule compounds screening

Screening results of potential drugs of GDM therapy were downloaded from CMap, ranking based on connectivity scores. The top 10 small molecule compounds identified as potential options for GDM treatment were penicillic-acid, lacidipine, YC-1, RITA, ALW-II-49-7, SA-792709, isoliquiritigenin, VX-222, CNQX and WH-4023, respectively (Table 3).

**TABLE 3:** Ten compounds identified as potential GDM therapeutics.

Chemical name	Chemical formula	Type	Score	Description
RITA	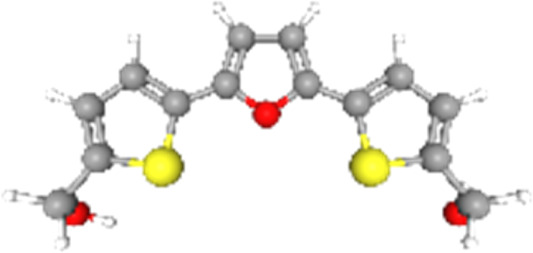	cp	−95.52	MDM inhibitor
penicillic-acid	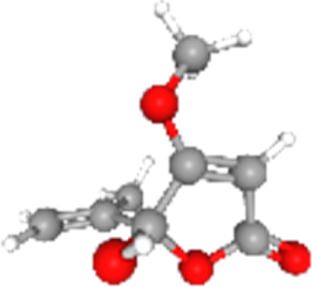	cp	−94.11	other antibiotic
isoliquiritigenin	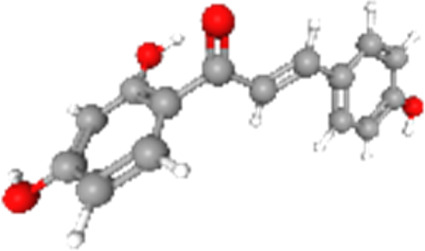	cp	−92.86	Guanylate cyclase activator
lacidipine	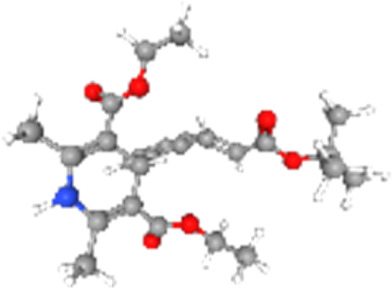	cp	−92.6	Calcium channel blocker
ALW-II-49–7	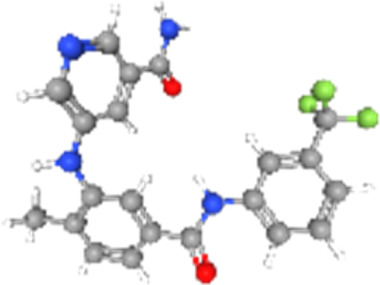	cp	−92.3	Ephrin inhibitor
SA-792709	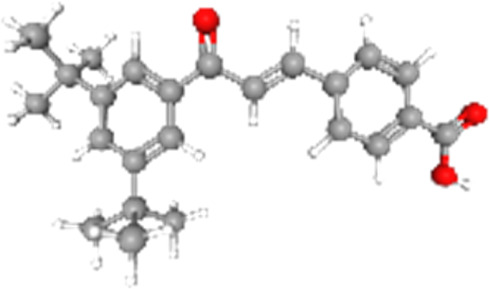	cp	−86.22	Retinoid receptor agonist
VX-222	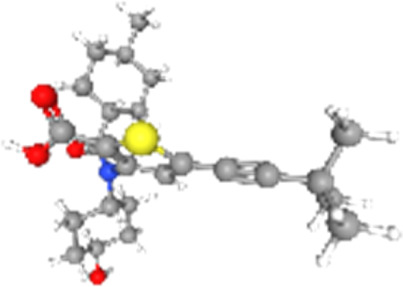	cp	−83.49	HCV inhibitor
WH-4023	—	cp	−83.19	SRC inhibitor
YC-1	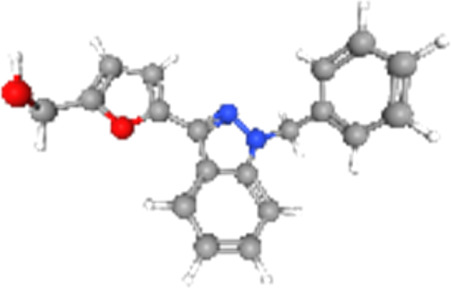	cp	−81.88	Guanylyl cyclase activator
CNQX	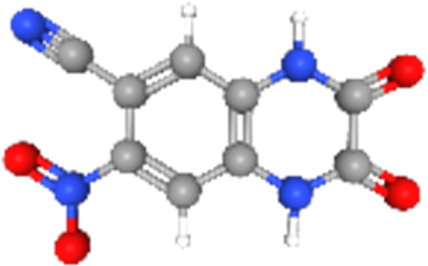	cp	−80.91	Glutamate receptor antagonist

Chemical formula were from Pubchem. cp denotes compound.

### Construction of hub genes network

**FIGURE 7:**
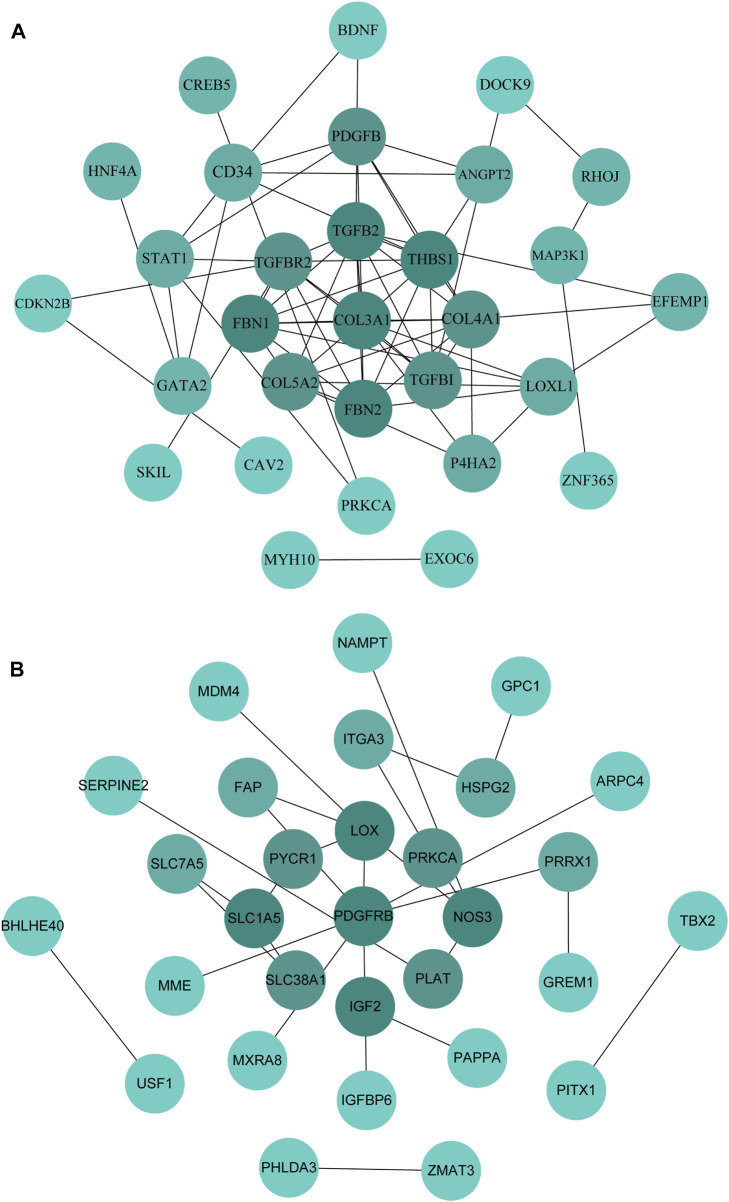
Identification of the hub genes in the PPI network. **(A)**: PPI network of the top 30 hub genes for upregulated genes. **(B)**: PPI network of the top 30 hub genes for downregulated genes.

The PPI network was constructed by STRING and displayed by Cytoscape to identify hub genes. The top 30 hub genes for upregulated and downregulated genes were detected by the Maximal Clique Centrality (MCC) of CytoHubba, respectively (shown in Figures 7A,B). Then, a total of the top 20 hub genes were selected to validate their expression level using external dataset (Table 4).

**TABLE 4:** Top 10 hub genes of the candidate genes in the PPI network ranked by MCC.

Up-regulated genes			Down-regulated genes		
Gene symbol	Score	miRNA	Gene symbol	Score	miRNA
COL3A1	469	miR-29b-3p	PDGFRB	7	miR-486-3p miR-6852-5p
TGFB2	436	miR-29b-1-5p miR-142-3p	LOX	5	miR-378a-5p
FBN2	387	miR-9-5p miR-212-5p	NOS3	4	miR-11401
FBN1	386	miR-542-3p miR-9-5p miR-508-3p	IGF2	3	miR-378a-5p miR-1908-5p
THBS1	319	miR-493-5p	SLC1A5	3	miR-6515-5p
COL4A1	298	miR-29b-3p	PLAT	2	-
COL5A2	156	miR-432-5p	SLC7A5	2	miR-486-3p
TGFBR2	147	miR-9-5p miR-181d-5p miR-301a-3p miR-432-5p	PYCR1	2	miR-378a-5p
TGFBI	49	miR-9-5p	SLC38A1	2	miR-6515-5p
PDGFB	33	miR-432-5p	FAP	2	miR-345-5p

miRNA: Corresponding miRNA.

### Validation of hub genes expression

In this study, through GEO dataset GSE103552, 20 hub genes were validated, including COL3A1, TGFB2, FBN2, FBN1, THBS1, COL4A1, COL5A2, TGFBR2, TGFBI, PDGFB (upregulated genes) and PDGFRB, LOX, NOS3, IGF2, SLC1A5, PLAT, SLC7A5, PYCR1, SLC38A1, FAP (downregulated genes).

**FIGURE 8:**
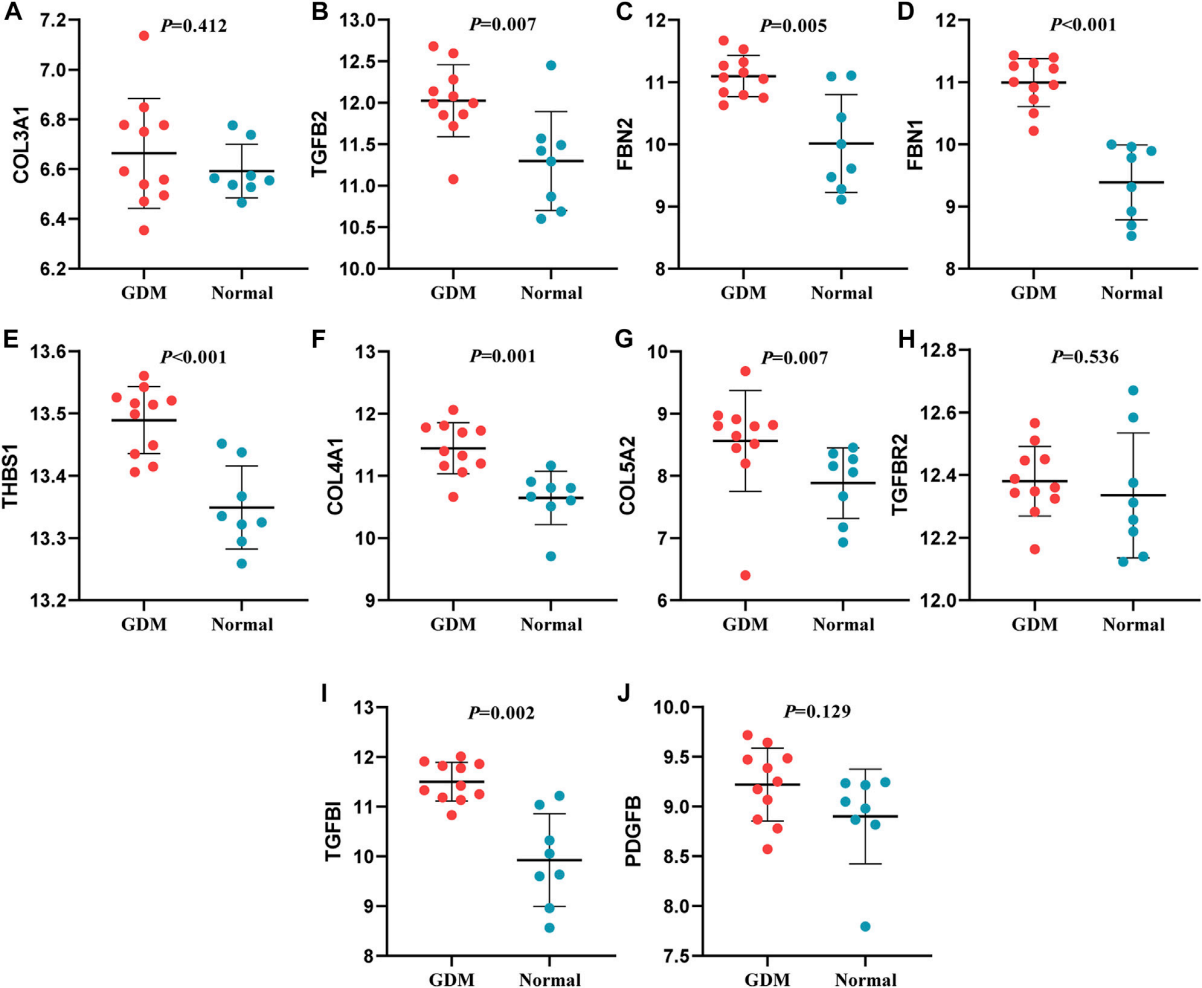
Verification of up-regulated genes. **(A)**: COL3A1 **(B)**: TGFB2 **(C)**: FBN2 **(D)**: FBN1 **(E)**: THBS1 **(F)**: COL4A1 **(G)**: COL5A2 **(H)**: TGFBR2 **(I)**: TGFBI **(J)**: PDGFB.

**FIGURE 9:**
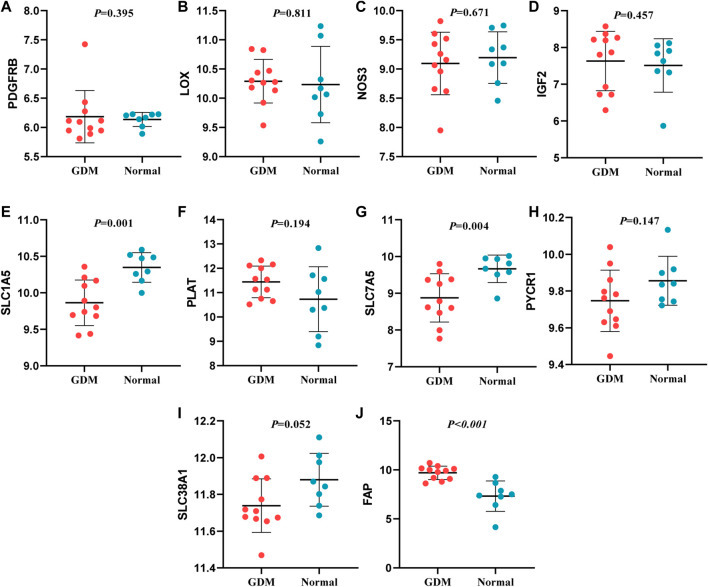
Verification of down-regulated genes. **(A)**: PDGFRB **(B)**: LOX **(C)**: NOS3 **(D)**: IGF2 **(E)**: SLC1A5 **(F)**: PLAT **(G)**: SLC7A5 **(H)**: PYCR1 **(I)**: SLC38A1 **(J)**: FAP.

The expression of TGFB2, FBN2, FBN1, THBS1, COL4A1, COL5A2 and TGFBI presented a similar trend of upregulation with the bioinformatic analysis; SLC7A5 and SLC1A5 expression were similar to the previous findings. Whereas, the expression of FAP was incompatible with previous screening result, and the expression of other mRNAs were not significantly different (*p* > 0.05) (Figures 8, 9). Therefore, miR-29b-1-5p-TGFB2, miR-142-3p-TGFB2, miR-9-5p-FBN2, miR-212-5p-FBN2, miR-542-3p-FBN1, miR-9-5p-FBN1, miR-508-3p-FBN1, miR-493-5p-THBS1, miR-29b-3p-COL4A1, miR-432-5p-COL5A2, miR-9-5p-TGFBI, miR-486-3p-SLC7A5 and miR-6515-5p-SLC1A5 were revealed as thirteen possible regulating pathways in our study.

## Discussion

As one of the most common complications of pregnancy, the biological mechanism of GDM remains to be clearly elucidated. Exploring the aetiology and progression of GDM, as well as supporting the development of disease-modifying treatments, is a clear and urgent need. miRNAs have potential function in essential biological activities and their dysregulation or dysfunction was revealed in metabolic researches regarding GDM, rendering them a potential role as biomarkers or therapeutic targets. By merging internal RNA-seq data and GEO datasets with integrated bioinformatic analysis, we were able to produce solid evidence to considerably intensify the likelihood of detecting candidate biological markers and tremendously improve the reliability of our findings. In our study, some core modules and critical signaling pathway were discovered and extracted from dysregulated ceRNA that shed a light on plausible etiology of GDM. Collectively, the present study highlighted the effect of dysregulated glycometabolism and hormone-related in GDM and revealed the complexity of miRNAs as important fine tune regulators in the biological processes and their potential as novel biomarkers and treatment targets in GDM.

Our study identified 193 candidate genes by overlapping DEGs and target genes of DEMs. The GO analysis exhibited that candidate genes were primarily involved in post-embryonic eye morphogenesis, paracrine signaling, sequestering of TGF-β in extracellular matrix, platelet-derived growth factor receptor-beta signaling pathway, melanocyte proliferation and glutamine transport. These result indicate that GDM is related to placental development and endocrine. Previously, a study reported that GDM could alter angiogenesis by modulating paracrine factors (Loegl et al., 2017). A study suggested that platelet derived growth factor receptor beta polypeptide (Pdgfrb) expression were decreased in maternal hyperglycemia compared to controls in animal model (Lehtoranta et al., 2016). Wang et al. found glutamine capacity significantly decreased in the fetuses of GDM (Wang et al., 2019). Interestingly, the upregulated genes were mostly found in TGF-β signaling pathway, AGE-RAGE signaling pathway in diabetic complications, Growth hormone synthesis, secretion and action and Pancreatic cancer *via* KEGG analysis, and these pathways were involved in inflammatory response. It suggests that inflammatory response are also involved in the pathology of GDM development.

Since there are no effective drugs for GDM, the online database was used to aid the prediction of some drugs. The analysis *via* CMap suggested that the top 10 small molecule compounds could have potential therapeutic effect on GDM. Isoliquiritigenin, as one of the most important chalcone compounds, presents the antidiabetic activity and plays a part in the suppression of inflammatory pathways (Gaur et al., 2014; Gupta et al., 2018; Zhao et al., 2019). Soluble guanylate cyclase (sGC) activator YC-1 mimicked Bradykinin (BK) enhance the uptake of insulin-stimulated glucose (Frigolet et al., 2017). However, more experimental studies are necessary to validate therapeutic effects of these potential drugs on GDM. And, their application to the clinical settings requires extensive basic research and clinical trials in the future.

The PPI network revealed the intercommunication of candidate miRNAs and target genes, among which 13 potential miRNA-target gene regulatory pathways were miR-29b-1-5p-TGFB2, miR-142-3p-TGFB2, miR-9-5p-FBN2, miR-212-5p-FBN2, miR-542-3p-FBN1, miR-9-5p-FBN1, miR-508-3p-FBN1, miR-493-5p-THBS1, miR-29b-3p-COL4A1, miR-432-5p-COL5A2, miR-9-5p-TGFBI, miR-486-3p-SLC7A5 and miR-6515-5p-SLC1A5, respectively. For those upregulated DEMs, plasma miR-486-3p was upregulated in T2DM patients compared to healthy controls (Meerson et al., 2019) and increased concentrations in prepubertal obesity (Prats-Puig et al., 2013), which indicate that miR-486-3p could participate in a metabolism-related mechanism of GDM. Among those downregulated DEMs, miR-9-5p was upregulated in endocrine pancreatic (EN) of pluripotent stem cells (hiPSCs) compared to undifferentiated hiPSCs, and the pancreas has a regulatory role in glycemia (Sebastiani et al., 2017). However, miR-9-5p had higher levels in GDM patients (Martínez-Ibarra et al., 2019). A study reported that miR-29b-3p expression level was decreased in mice obesity model induced by high-fat diet (Zhang et al., 2022), while miR-29b-3p showed higher levels in the early phase of placentation in GDM(6–15 weeks of gestation) (Gillet et al., 2019). These might be related to the differences of sample source and gestation periods. Additionally, miR-542-3p, miR-493-5p, miR-432-5p and miR-6515-5p were reported to participate in the progression of breast cancer (Jiang et al., 2022), diabetic osteoporosis (Zhai et al., 2020), pancreatic ductal adenocarcinoma (Shen et al., 2021), inflammation-related disorders (periodontitis) (Xu et al., 2020). Those critical miRNAs, in particular, play significant roles in regulating biological processes in GDM.

In our study, interestingly, the expression of 9 genes (TGFB2, FBN2, FBN1, THBS1, COL4A1, COL5A2, TGFBI, SLC7A5, SLC1A5), being consistent with the result in GSE103552, were further verified. TGFB2 encodes a secreted ligand of the transforming growth factor-beta (TGF-β) superfamily of proteins, which is involved in the pathogenesis of diabetes and complications. Zhou et al. reported that TGFB2 is an upregulated gene in the plasma of GDM patients and correlated with FBG levels in GDM patients (Zhou et al., 2021), which suggested that TGFB2 might play vital roles in the pathogenesis of GDM. FBN2 encodes a peptide hormone placensin, which stimulates cAMP-PKA signaling, glucose secretion and trophoblast invasion in human trophoblastic cells. During third trimester, serum placensin levels of GDM patients are increased to a bigger extent compared to healthy pregnant women (Yu et al., 2020). FBN1 can encode the protein hormone asprosin, which is involved in regulating glucose homeostasis (Goodarzi et al., 2021). Zhong et al. reported that asprosin was highly expressed in the plasma of GDM patients and their offspring (Zhong et al., 2020). The protein encoded by THBS1 is an adhesive glycoprotein and proinflammatory cytokine that promotes insulin resistance. A study suggested that THBS1 in obesity or type 2 diabetes mellitus was highly expressed than healthy controls (Tang et al., 2020). COL4A1 was involved in diabetic tubulointerstitial injury (Zeng et al., 2019) and COL5A2 participated in the progression of uterine fibroids (Giri et al., 2017) and colon adenocarcinoma (Wei et al., 2020), although their functions were not fully understood for GDM development. TGFBI, upregulated in the placenta and the plasma of GDM, was positively associated with FBG levels in GDM patients (Han et al., 2014; Zhou et al., 2021). SLC7A5 enables L-leucine/L-tryptophan transmembrane transporter activity and is a part of amino acid transport complex. One study reported the uptake of ^14^C-l-methionine by human trophoblasts derived from normal pregnancies is mainly mediated by L-type amino acid transporter 1 [LAT1 (L), SLC7A5]. However, the process exposed to the high glucose environment of GDM may alter the nature of transporters involved in the uptake process (Araújo et al., 2013). The study of HOLM et al. reported that SLC1A5 was involved in sphingolipid metabolism that contributes to genetic predisposition to type 1 diabetes, whereas there has been no direct evidence of the association between SLC1A5 and GDM risk (Holm et al., 2018).

Our study has obvious merits. We have systematically analyzed the miRNAs expression and their target genes between 6 GDM and 6 healthy controls integrating with public GEO dataset. The sample size of the integrated analysis is adequate to explore and verify those candidate miRNAs and key genes. Furthermore, we investigated the biological functions and potential therapeutic small molecule compounds of miRNA-target genes, as well as obtained pivotal genes through bioinformatics analysis. More importantly, when being compared with placenta, collecting blood samples is actually more accessible in clinical practice as early screening and diagnosis.

Also, there are some limitations that need be considered. First, due to the availability of GSE103552, our study did not analyze the association between clinical data (clinical parameters and prognosis) and genetic change. Additionally, as the statistical power based on its relatively larger sample size, GSE103552 were chosen as the external validation dataset. Second, we acknowledge the relatively low sample size of internal screening dataset as limitation of our study. We integrated internal samples and the 3 diverse public databases to comprehensively screen the critical miRNAs and key genes, and this could present a comprehensive screening results and predictions. However, external experimental verifications are further needed to elucidate the molecular mechanisms of GDM in the future.

In conclusion, this study demonstrated a series of aberrantly differentially expressed miRNAs and genes that are associated with epigenetic alternations of miRNAs in GDM by overlapping DEGs and targets of DEMs. A multitude of novel miRNA-targeting genes and regulatory pathways were identified that might serve as high priority targets for therapeutic interventions (miR-29b-1-5p-TGFB2, miR-142-3p-TGFB2, miR-9-5p-FBN2, miR-212-5p-FBN2, miR-542-3p-FBN1, miR-9-5p-FBN1, miR-508-3p-FBN1, miR-493-5p-THBS1, miR-29b-3p-COL4A1, miR-432-5p-COL5A2, miR-9-5p-TGFBI, miR-486-3p-SLC7A5 and miR-6515-5p-SLC1A5). Furthermore, ten chemicals were identified as putative therapeutic agents for GDM. Precise diagnosis and therapeutic targets of GDM would be further explored through putative genes in the future.

## Data Availability

The original contributions presented in the study are publicly available. This data can be found here: https://www.ncbi.nlm.nih.gov/geo/ accession number: GSE218696.

## Abbreviations

CMap, connectivity map; DEGs, differentially expressed genes; DEMs, differentially expressed microRNAs; GDM, gestational diabetes mellitus; GEO, gene expression omnibus; GO, gene ontology; GWAS, genome-wide association studies; KEGG, kyoto encyclopedia of genes and genomes; miRNA, MicroRNA; PBL, peripheral blood leukocytes; PPI, protein-protein interaction.
